# Associations Between Potassium Channel Genes and the Occurrence of Palpitations in Women Prior to Breast Cancer Surgery

**DOI:** 10.1016/j.soncn.2025.152039

**Published:** 2025-10-15

**Authors:** Ying Sheng, Matthew R. Fleming, Carolyn J. Harris, Jon D. Levine, Steven M. Paul, Janet S. Carpenter, Yvette P. Conley, Joosun Shin, Kate Oppegaard, Marilyn J. Hammer, Christine Miaskowski

**Affiliations:** a School of Nursing, Vanderbilt University, Nashville, Tennessee; b Department of Cardiovascular Medicine, Vanderbilt University Medical Center, Nashville, Tennessee; c School of Nursing, University of California, San Francisco, California; d School of Medicine, University of California, San Francisco, California; e School of Nursing, Indiana University, Indianapolis, Indianna; f School of Nursing, University of Pittsburgh, Pittsburgh, Pennsylvania; g School of Nursing, University of California, Los Angeles, Los Angeles, California; h Department of Nursing, VA Portland Health Care System, Portland, Oregon; i Phyllis F. Cantor Center, Dana Farber Cancer Institute, Boston, Massachusetts

**Keywords:** Breast cancer, Cardiac toxicity, Genetics, Genomics, Palpitations, Polymorphism, Potassium channels

## Abstract

**Objectives:**

The aim of the study was to evaluate for associations between the occurrence of palpitations in women prior to breast cancer surgery and single nucleotide polymorphisms (SNPs) for potassium channel genes.

**Methods:**

A total of 398 women were recruited prior to breast cancer surgery and provided detailed information on demographic and clinical characteristics. The occurrence of palpitations was assessed using a single item (ie, did you experience your “heart race/pounds” in the past week – yes or no). Blood samples were collected for genomic analyses and genotyping of single nucleotide polymorphisms (SNPs) was done using a custom array. Multiple logistic regression analyses were used to identify associations between the occurrence of palpitations and variations in potassium channel genes.

**Results:**

After controlling for functional status and the occurrence of back pain, significant associations were found between the occurrence of palpitations and six SNPs among five candidate genes, including potassium voltage-gated channels (ie, potassium voltage gated channel modifier subfamily S member 1 (*KCNS1*) rs4499491), potassium inwardly rectifying channels (ie, potassium inwardly rectifying channel subfamily J member 3 (*KCNJ3*) rs717175, *KCNJ* subfamily J member 5 (*KCNJ5*) rs11221510, *KCNJ* subfamily J member 6 (*KCNJ6*) rs13049947 and *KCNJ6* rs1399596), and potassium two pore domain channels (ie, potassium two pore domain channel subfamily K member 2 (*KCNK2*) rs12757222).

**Conclusions:**

Variations in potassium channel genes are associated with the occurrence of palpitations in women prior to breast cancer surgery.

**Implications for Nursing Practice:**

While direct clinical implications cannot be made, these findings provide preliminary evidence of potential therapeutic targets.

## Introduction

Over the last decade, evidence suggests that 15% to 48% of women with breast cancer experience the distressing cardiac sensation of their heart “pounding or racing,” referred to as a “palpitation.”^1–3^ This range in prevalence rates is wider than the rates of 42% and 54% reported by healthy peri- and postmenopausal women, respectively.^4^ In patients with cardiovascular disease, palpitations are the second leading reason for a visit to a cardiologist.^5^ Of note, in cancer patients, cardiovascular disease is the second leading cause of morbidity and mortality.^6^ In fact, compared to the general population, cancer patients have a 3.93-fold increased risk of mortality from cardiovascular disease that persists long into survivorship.^7^

In a scoping review of 84 studies that investigated risk factors for palpitations in menopausal women,^8^ only three^9–11^ evaluated for biomarkers. While in one study,^11^ no associations were found, in the other two studies,^9,10^ the occurrence of palpitations was associated with different polymorphisms in the cytochrome P450 family 1 subfamily B member 1 gene (*CYP1B1*). This gene catalyzes numerous reactions including the synthesis of cholesterol and other steroids including estradiol. In terms of women with breast cancer, recent work from our group suggested that palpitations were associated with variations in genes involved in inflammation (eg, interleukin (IL) 1-beta (*IL1B*), *IL10*, *IL13*)^12^ and neurotransmission (eg, serotonergic, catecholaminergic, dopamine, and gabaergic).^13^ These findings suggest that genetic mechanisms may contribute to the occurrence of palpitations in women with and without breast cancer. However, additional studies are warranted to determine underlying mechanisms for this common clinical condition.

Potassium channels play a critical role in cardiac function by regulating the flow of potassium ions across the membranes of cardiac muscle cells.^14,15^ While several types of potassium channels exist (eg, voltage-gated, inwardly rectifying, two pore domain channels), their main function is to regulate repolarization and maintain the resting membrane potential of cardiac cells.^16^ Variations in potassium channel genes are associated with atrial fibrillation,^17^ heart failure,^18^ and long QT syndrome.^19–21^ Of note, patients with these conditions may report the occurrence of palpitations. However, no studies were identified that documented associations between palpitations and polymorphisms in potassium channel genes in patients with breast cancer. Therefore, the purpose of this study was to investigate associations between the occurrence of palpitations in women prior to breast cancer surgery and single nucleotide polymorphisms (SNPs) for potassium channel genes. Given their important role in cardiac function, the identification of associations between the occurrence of palpitations and genetic variations in potassium channel genes may be useful to identify patients at increased risk for this troublesome symptom and associated cardiovascular conditions.

## Methods

### Sample and Settings

This genomic analysis draws its data from a larger, longitudinal study that evaluated neuropathic pain and lymphedema in women following breast cancer surgery. Details of the parent study’s methods are published elsewhere.^22^ The Theory of Symptom Management (symptom, person, clinical, and biological factors) guided this study.^23^ Patients were recruited from breast care centers located in a Comprehensive Cancer Center, two public hospitals, and four community practices. Inclusion criteria were women who were ≥18 years of age, scheduled for unilateral breast cancer surgery, able to read, write, and speak English, and provided written informed consent. Exclusion criteria were scheduled for bilateral breast cancer surgery or distant metastasis at the time of diagnosis. A total of 516 patients were approached; 410 were enrolled (response rate 79.5%); 398 completed the enrollment assessment; and 310 provided a blood sample for genetic analysis. Commonly cited reasons for refusal to participate were too busy, overwhelmed with the cancer diagnosis, or insufficient time to complete the enrollment assessment prior to surgery.

The study was approved by the Committee on Human Research at the University of California, San Francisco and the Institutional Review Boards at each of the study sites. During the patient’s preoperative visit, a clinician explained the study to the patient and determined her willingness to participate. For those women who were willing to participate, the clinician introduced the patient to the research nurse who determined eligibility and obtained written informed consent. After obtaining informed consent, patients completed the enrollment questionnaire and provided a blood sample an average of four days prior to surgery.

### Measures

Patients completed self-report questionnaires to capture demographic and clinical characteristics. Comorbidity burden was measured using the Self-Administered Comorbidity Questionnaire (SCQ).^24,25^ Functional status was assessed with the Karnofsky Performance Status (KPS) scale.^26^ Medical records were reviewed to obtain details on disease status and treatment history.

The occurrence of palpitations was evaluated using a single item from the Menopausal Symptoms Scale, adapted from the Seattle Women’s Health Study questionnaire.^27^ Women were asked whether they had experienced their “heart races/pounds” in the past week. This single-item self-report approach aligns with prior studies assessing palpitations in women with^1,2^ and in over 100 studies of women without^28^ breast cancer.

### Candidate Gene Selection and Genotyping

#### Blood collection and genotyping –

Genomic deoxyribonucleic acid (DNA) was extracted from peripheral blood mononuclear cells using the PUREGene DNA Isolation System (Invitrogen). Samples were genotyped using the Golden Gate genotyping platform (Illumina) and processed according to the standard protocol using GenomeStudio (Illumina).

#### SNP selection –

Combination of tagging SNPs and literature-driven SNPs were selected for analysis. Tagging SNPs (ie, a representative SNP within a region of the gene that had high linkage disequilibrium and can serve as a marker for other SNPs in the same region) were required to be common (defined as having a minor allele frequency of ≥.05) in public databases (eg, HapMap). In order to ensure robust genetic association analyses, quality control filtering of SNPs was performed. SNPs with call rates of <95% or a Hardy-Weinberg *p*-value of <.001 were excluded.

As shown in Supplementary Table 1, a total of 155 SNPs among the 10 candidate genes (ie, potassium voltage-gated channel subfamily A member 1 (*KCNA1*): 1 SNP; potassium voltage-gated channel subfamily D member 2 (*KCND2*): 9 SNPs; potassium voltage-gated channel modifier subfamily S member 1 (*KCNS1*): 4 SNPs; potassium inwardly rectifying channel subfamily J member 3 (*KCNJ3*): 28 SNPs; potassium inwardly rectifying channel subfamily J member 5 (*KCNJ5*): 8 SNPs; potassium inwardly rectifying channel subfamily J member 6 (*KCNJ6*): 58 SNPs; potassium inwardly rectifying channel subfamily J member 9 (*KCNJ9*): 2 SNPs; potassium two pore domain channel subfamily K member 2 (*KCNK2*): 22 SNPs; potassium two pore domain channel subfamily K member 3 (*KCNK3*): 6 SNPs; potassium two pore domain channel subfamily K member 9 (*KCNK9*): 17 SNPs) passed all of the quality control filters and were included in the genetic association analyses.

All genes were identified according to the approved symbol stored in the Human Genome Organization Gene Nomenclature Committee database (http://www.genenames.org). Function of the genes was determined using GeneCard.^29,30^ Localization of SNPs and regional annotations were identified using the University of California Santa Cruz Genome Browser for the human reference assembly GRCh38/hg38 (http://genome.ucsc.edu). Potential regulatory involvement of SNPs was investigated using SNPinfo.^31^ Potential functional roles for SNPs were investigated using annotation data from the Encyclopedia of DNA elements (ENCODE)^32^ and expression quantitative trait loci (eQTL) data from the Genome-Tissue Expression Project Portal.^33^ Linkage disequilibrium (LD) with other SNPs and/or eQTLs were evaluated using data from the 1000 Genomes Project with LDlink (https://ldlink.nci.nih.gov).^34^

### Statistical Analyses for Genetic Data

Allele and genotype frequencies were determined by gene counting. Hardy-Weinberg equilibrium was assessed using Chi-square or Fisher’s exact tests. For the haplotype determinations, measures of LD (ie, D’ and r^2^) were computed from the patients’ genotypes with Haploview 4.2. LD-based haplotype block definition was based on D’ confidence interval.^35^ For SNPs that were members of the same haploblock, haplotypes were constructed using the program PHASE version 2.1.^36^ Ancestry informative markers (AIMs) were used to minimize confounding due to population substructure.^37^

For association tests, three genetic models were assessed for each SNP: additive, dominant, and recessive using Chi-square or Fisher’s exact tests. For the significant SNPs, the genetic model that best fit the data, by maximizing the significance of the *p*-value was selected for that SNP. Logistic regression analyses, that controlled for significant covariates, as well as genomic estimates of and self-reported race and ethnicity, were used to evaluate the association between SNPs and haplotypes that were significant in bivariate analyses (Supplementary Table 1) and the occurrence of palpitations. A backwards stepwise approach was used to create the most parsimonious model. Except for genomic estimates of and self-reported race and ethnicity, only predictors with a *p*-value of <.05 were retained in the final model. Genetic model fit and both unadjusted and covariate-adjusted odds ratios were estimated using Stata Version 15.^38^

## Results

### Demographic and Clinical Characteristics

Our previous study provided comprehensive information on differences in demographic and clinical characteristics between the patients with and without palpitations.^3^ A summary of sample characteristics and between group differences are presented in Supplementary Table 2. In summary, the characteristics associated with the occurrence of palpitations included lower annual household income, decreased functional status, a higher comorbidity burden, and an increased likelihood of self-reporting a diagnosis of back pain.

### Candidate Gene Analysis

As shown in Table 1, 15 SNPs and six haplotypes among seven potassium channel genes were associated with the occurrence of palpitations: 2 SNPs and 1 haplotype in *KCND2*; 2 SNPs and 1 haplotype in *KCNS1*; 2 SNPs and 1 haplotype in *KCNJ3*; 2 SNPs in *KCNJ5*; 6 SNPs and 2 haplotypes in *KCNJ6*; 1 SNP in *KCNK2*; and 1 haplotype in *KCNK9*.

### Regression Analyses

To better estimate the magnitude (ie, odds ratio [OR]) and precision (ie, confidence interval [CI]) of genotype on the odds of reporting palpitations prior to breast cancer surgery, multivariable logistic regression models were fit. Using the backward stepwise approach, only KPS scores in 10 unit increments (functional status) and the occurrence of back pain remained significant in the logistic regression model.^12^ These characteristics were included as covariates in subsequent models that evaluated for associations between palpitations and polymorphisms in potassium channel genes. After controlling for KPS score, the occurrence of back pain, self-reported and genomic estimates of race and ethnicity, and variations in other SNPs and haplotypes within the same gene, six SNPs among five candidate genes were associated with the occurrence of palpitations (Table 2).

### Potassium Voltage-Gated Channels

**FIG 1.**
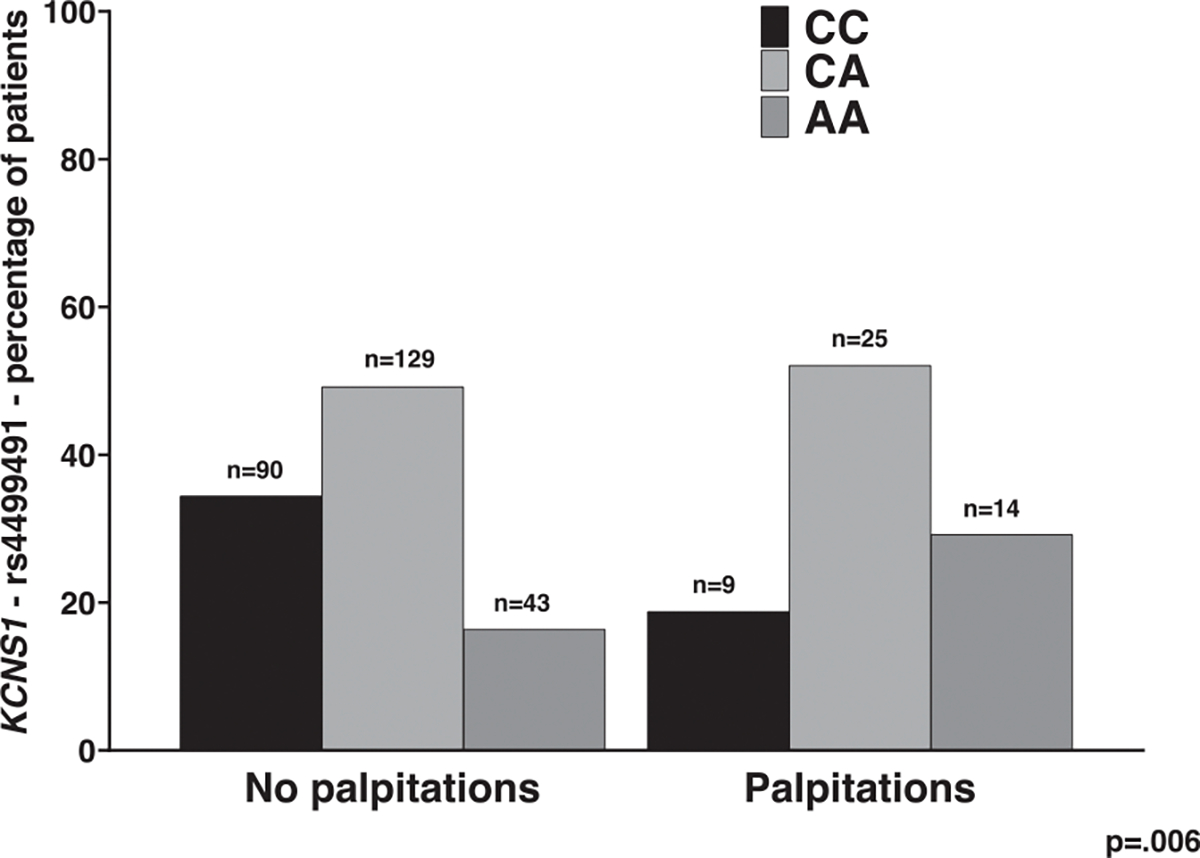
Differences between the palpitations groups in the percentages of patients who were homozygous for the common allele (CC) or heterozygous (CA) or homozygous (AA) for the rare allele for rs4499491 in potassium voltage-gated channel modifier subfamily S member 1 (*KCNS1*).

For *KCNS1* rs4499491, each additional dose of rare allele (CC versus CA versus AA) was associated with a 2.03 increase in the odds of belonging to the palpitations group (Figure 1).

### Potassium Inwardly Rectifying Channels

**FIG 2.**
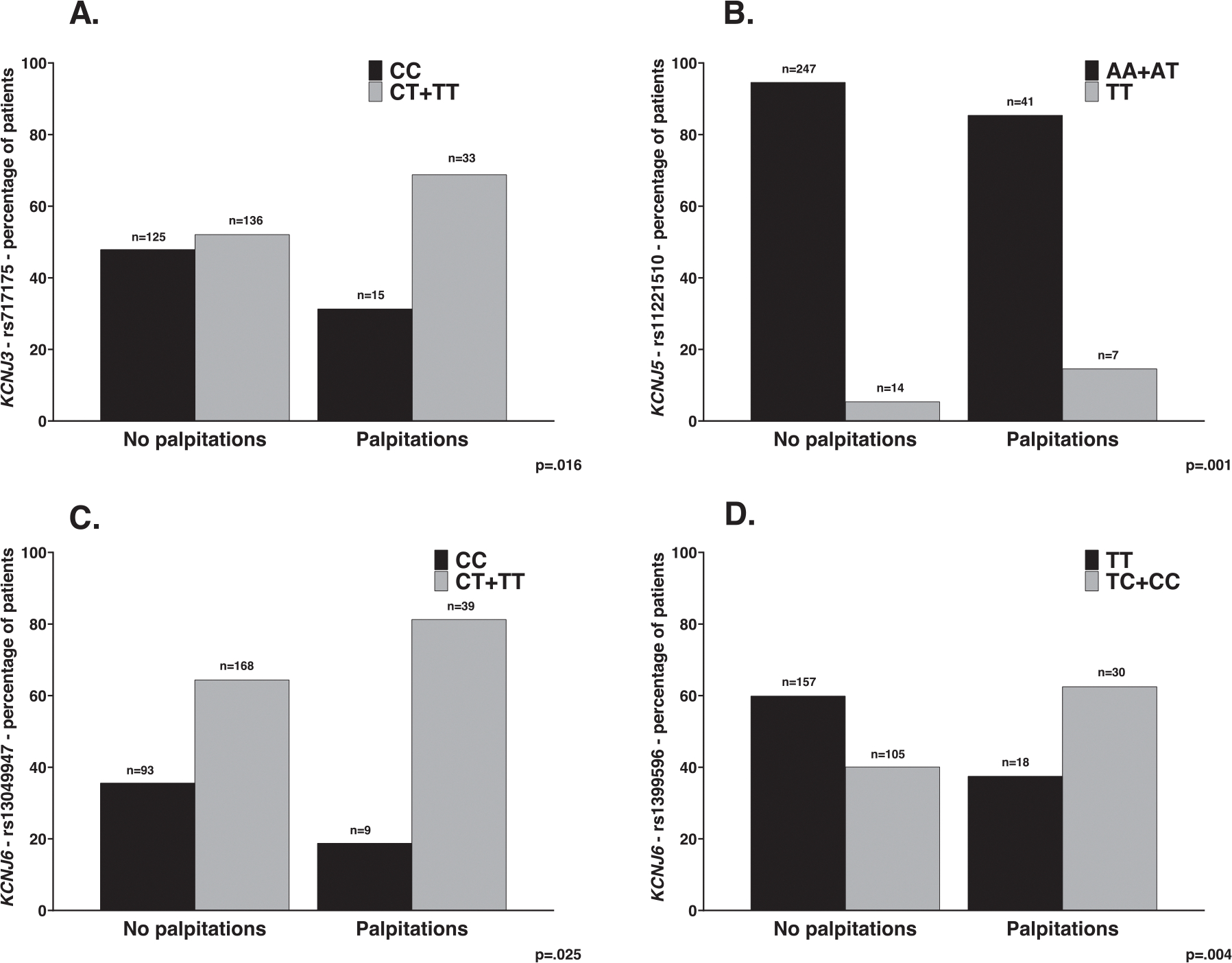
(A) Differences between the palpitations groups in the percentages of patients who were homozygous for the common allele (CC) or heterozygous or homozygous for the rare allele (CT+TT) for rs717175 in potassium inwardly rectifying channel subfamily J member 3 (*KCNJ3*). (B) Differences between the palpitations group in the percentages of patients who were who were homozygous or heterozygous for the common allele (AA+AT) or homozygous for the rare allele (TT) for rs11221510 in potassium inwardly rectifying channel subfamily J member 5 (*KCNJ5*). (C) Differences between the palpitations groups in the percentages of patients who were homozygous for the common allele (CC) or heterozygous or homozygous for the rare allele (CT+TT) for rs13049947 in potassium inwardly rectifying channel subfamily J member 6 (*KCNJ6*). (D) Differences between the palpitations groups in the percentages of patients who were homozygous for the common allele (TT) or heterozygous or homozygous for the rare allele (TC+CC) for rs1399596 in potassium inwardly rectifying channel subfamily J member 6 (*KCNJ6*).

For *KCNJ3* rs717175, carrying one or two doses of rare allele (CC versus CT+TT) was associated with a 2.49 increase in the odds of belonging to the palpitations group (Figure 2A). For *KCNJ5* rs11221510, carrying two doses of rare allele (AA+AT versus TT) was associated with a 5.68 increase in the odds of belonging to the palpitations group (Figure 2B). For *KCNJ6*, two SNPs were associated with the occurrence of palpitations. For *KCNJ6* rs13049947, carrying one or two doses of rare allele (CC versus CT+TT) was associated with a 2.73 increase in the odds of belonging to the palpitations group (Figure 2C). In the same regression analysis, for *KCNJ6* rs1399596, carrying one of two doses of rare allele (TT versus TC+CC) was associated with a 2.83 increase in the odds of belonging to the palpitations group (Figure 2D).

### Potassium Two Pore Domain Channels

**FIG 3.**
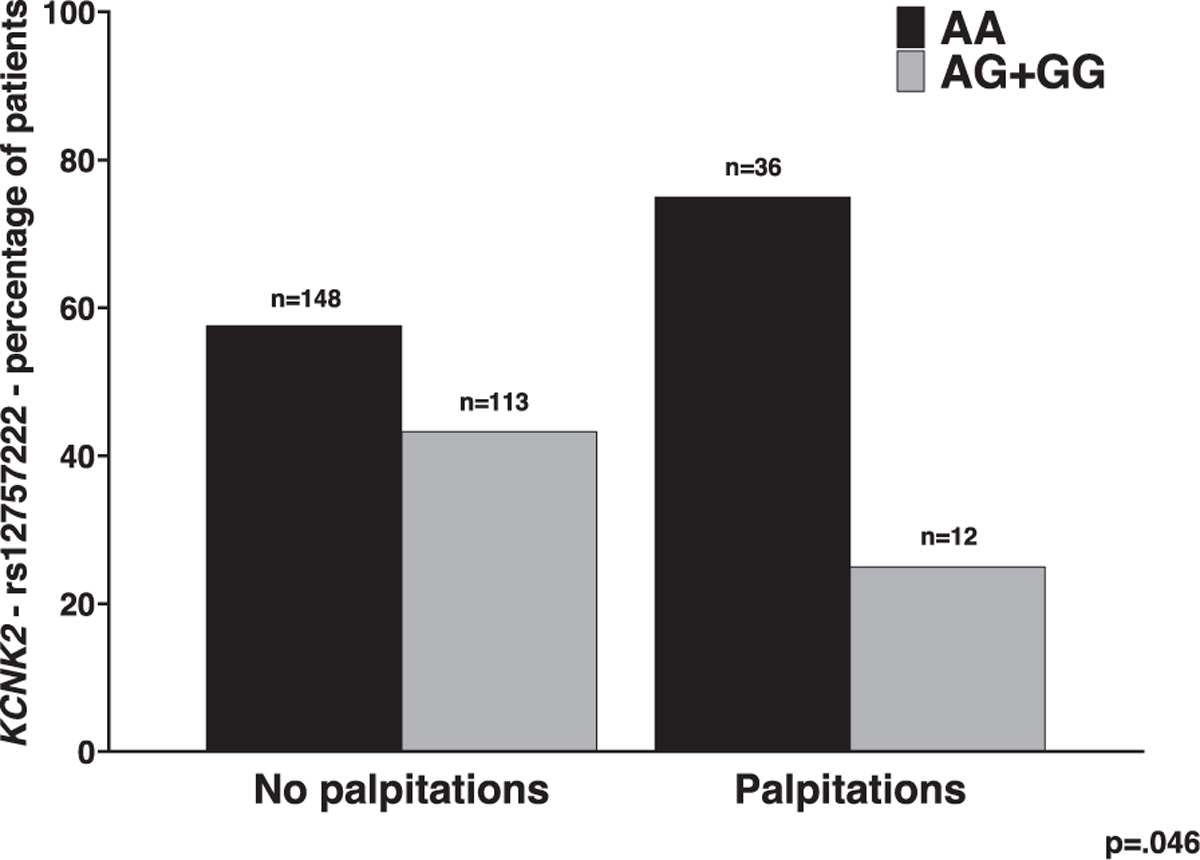
Differences between the palpitations groups in the percentages of patients who were homozygous for the common allele (AA) or heterozygous or homozygous for the rare allele (AG+GG) for rs12757222 in potassium two pore domain channel subfamily K member 2 (*KCNK2*).

For *KCNK2* rs12757222, carrying one or two doses of rare allele (AA versus AG+GG) was associated with a 55% decrease in the odds of belonging to the palpitations group (Figure 3).

## Discussion

This study is the first to investigate the associations between the occurrence of palpitations and potassium channel genes in women prior to breast cancer surgery. Of the six significant SNPs in this study, one was associated with potassium voltage-gated channel genes (ie, *KCNS1* rs4499491); four with potassium inwardly rectifying channel genes (ie, *KCNJ3* rs717175, *KCNJ5* rs11221510, *KCNJ6* rs13049947, *KCNJ6* rs1399596), and one with a potassium two pore domain channel gene (ie, *KCNK2* rs12757222). Except for the SNP for *KCNS1*, all of the other SNPs are intron variants with no known function. While intron variants were originally thought to be non-coding and non-functional, evidence suggests that intron splicing is linked to the enhancement of transcription.^39^ Of note, *KCNS1* rs4499491 is located in the three prime untranslated region (3’-UTR) of the gene.^40^ This region contains elements that can influence messenger ribonucleic acid (mRNA) localization, stability, and translation.^41^ Therefore, this SNP may alter the function of potassium voltage-gated channels.^42^

In the heart, approximately 10 distinct potassium channels are involved in shaping the cardiac action potential. Dysfunction in these channels affects intracellular signaling, metabolism, remodeling, and arrhythmogenesis in several cardiovascular disorders.^43^ Of note, the interplay between different potassium channels in both the atria and the ventricles is dynamic. Any posttranscriptional and posttranslational remodeling of individual potassium channels changes their activity relative to each other and can result in serious arrhythmias.^44^

### Potassium Voltage-Gated Channel Genes

Potassium voltage-gated channels (Kvs) form the largest and most diversified class of ion channels. Their main functions include regulation of resting potential of the cell membrane, as well as control of the shape and frequency of action potentials.^43,45^ Their proper functioning is essential for the maintenance of normal cardiac activity.^46,47^

*KCNS1* belongs to the potassium voltage-gated channel subfamily S, member 1 that encodes Kv9.1 and modulates neuronal excitability.^43,45^ While no preclinical or clinical evidence supports an association between palpitations and *KCNS1* rs4499491, in our previous study with the same sample,^48^ this SNP was associated with shortness of breath.

### Potassium Inwardly Rectifying Channels Genes

Potassium inwardly rectifying channels or G-protein-gated inwardly rectifying potassium (GIRK) channels are located in atrial and ventricular myocytes and play a major role in the regulation of heart neuronal excitability.^49,50^ Given this role, imbalances in this channel can lead to disturbances in cardiac rhythm.

*KCNJ3* encodes the GIRK channel subunit 1 (GIRK1) that is expressed in both the heart (ie, atria and sinoatrial node) and the brain.^50^ This channel combines with the GIRK channel subunit 4 (GIRK4) (ie, encoded by *KCNJ5*) to form the acetylcholine-activated potassium channel which is primarily expressed in the atrium.^50,51^ Evidence suggests that this channel is associated with bradyarrhythmias and atrial fibrillation.^51^

*KCNJ5* encodes the GIRK4, which is expressed in the atria and sinoatrial node.^50,52^ In addition, this gene is expressed in the paraventricular nucleus of the hypothalamus which is a region of the brain that controls cardiac vagal neuronal activation.^50^ As noted in one review,^52^ variations in *KCNJ5* are associated with familial hyperaldosteronism type III, long QT Syndrome, atrial fibrillation, and sinus node dysfunction. While no association between palpitations and *KCNJ5* rs717175 was reported in this review,^52^ in a genome-wide association study of the UK Biobank,^53^ cohort variations in this gene were associated with heart rate variability.

*KCNJ6* encodes GIRK channel subunit 2 (GIRK2), which regulates cardiac cellular and neuronal excitability by mediating the inhibitory effects of G protein-coupled receptors, essential for neuromodulation and neurotransmission.^54^ These channels are widely expressed throughout the heart and the brain. While evidence supports a role for *KCNJ6* gene variations in pain^55,56^ and Down syndrome,^54^ neither of the SNPs identified in the current study (ie, *KCNJ6* rs13049947, *KCNJ6* rs1399596) were associated with palpitations or other cardiac conditions. However, preliminary evidence suggests associations between variations in this gene and age-related decreases in cardiac function,^57^ aortic stenosis,^58^ and atrial fibrillation associated with valvular heart disease.^59^

### Potassium Two Pore Domain Channel Genes

Potassium two pore domain channels, known as K_2P_ channels regulate resting membrane potential and cellular excitability through outward potassium currents. Members of this family are expressed in various organs and regulate a wide variety of physiological processes.^15^ The *KCNK2* gene, also known as TREK-1, encodes the two pore domain subfamily K, member 2 channel.

TREK-1 is the most abundant two pore potassium channel in the human heart. These channels modulate cardiac repolarization. Alterations in these channels may be involved in cardiac hypertrophy, cardiac fibrosis, and heart failure.^15,60^ In addition, TREK-1 channels are implicated in the development of arrhythmias. For example, in a study of patients with genetically unresolved arrhythmia syndromes,^61^ a single nucleotide variant for *KCNK2* was associated with sudden and recurrent episodes of ventricular tachycardia. In addition, atrial TREK-1 mRNA levels are reduced in patients with atrial fibrillation and heart failure.^62,63^ Given the recent availability of TREK-1 inhibitors and activators, this channel may become a therapeutic target to control cardiac rhythm disturbances and heart failure.^60^ In fact, in a preclinical gene therapy study of pigs with atrial fibrillation,^62^ gene transfer increased TREK-1 protein levels and attenuated the prolongation of the atrial effective refractory period. Given the associations between arrhythmias and palpitations,^64,65^ additional research is warranted on the role of TREK-1 channels.

### Limitations

Several limitations need to be acknowledged. Given its cross-sectional design, longitudinal studies are needed to evaluate the causal relationships between ongoing occurrence and severity of palpitations and variations for potassium channel genes. A summary of sample characteristics and between group differences are presented in Supplemental Table 2. In addition, because a candidate gene approach was used, additional polymorphisms, as well as other potassium channel genes, warrant evaluation. Given that patients were primarily White, well-educated and had a diagnosis of breast cancer, replication is warranted in more diverse samples with various types of cancer that affect both men and women to increase the generalizability of the findings. Since the sample size was relatively small, additional studies are needed to confirm these findings.

## Conclusions

This study provides preliminary evidence for associations between the occurrence of palpitations and variations in genes that code for potassium voltage-gated channels, potassium inwardly rectifying channels, and potassium two pore domain channels in women undergoing surgery for breast cancer. While direct clinical implications cannot be made because of the exploratory nature of this study, future preclinical and clinical studies are needed to determine the underlying mechanisms for these associations. Additional research may lead to the development and testing of interventions to prevent or treat this symptom and result in decreases in morbidity and mortality associated with cardiovascular disease.

## Supplementary Material

1

2

Supplementary material associated with this article can be found in the online version at doi:10.1016/j.soncn.2025.152039.

## Figures and Tables

**TABLE 1:** Summary of Single Nucleotide Polymorphisms Analyzed for Potassium Channel Genes That Demonstrated Significant Bivariate Associations With the Occurrence of Palpitations Prior to Breast Cancer Surgery

Gene	SNP	Position	Chr	MAF	Alleles	Chi Square	*p*-value	Model

POTASSIUM VOLTAGE-GATED CHANNELS								
KCND2	rs4730967	120060462	7	0.320	T>C	FE	.039	R
KCND2	rs11489533	120117902	7	0.268	A>G	12.364	.002	A
KCND2	HapA1					12.435	.002	
KCNS1	rs4499491	43154833	20	0.432	C>A	6.747	.034	A
KCNS1	rs6124684	43154907	20	0.223	C>T	FE	.036	D
KCNS1	HapA1					6.748	.034	
Potassium inwardly rectifying channels								
KCNJ3	rs13398937	155348593	2	0.362	C>G	FE	.038	D
KCNJ3	rs717175	155356841	2	0.332	C>T	FE	.040	D
KCNJ3	HapA1					7.113	.029	
KCNJ5	rs11221503	128277662	11	0.184	C>T	FE	.049	R
KCNJ5	rs11221510	128285907	11	0.241	A>T	FE	.029	R
KCNJ6	rs13049947	38002710	21	0.403	C>T	FE	.029	D
KCNJ6	rs858035	38021061	21	0.344	T>C	FE	.017	D
KCNJ6	rs13048511	38037731	21	0.468	A>G	FE	.044	R
KCNJ6	rs857989	38042001	21	0.115	G>C	6.146	.046	A
KCNJ6	rs1399596	38045382	21	0.260	T>C	FE	.004	D
KCNJ6	rs2154556	38120757	21	0.344	T>C	FE	.025	R
KCNJ6	HapD1					6.364	.042	
KCNJ6	HapE1					8.793	.012	
Potassium Two Pore Domain Channels								
KCNK2	rs12757222	213391641	1	0.233	A>G	FE	.024	D
KCNK9	HapD1					6.916	.031	

A = additive model; Chr = chromosome; D = dominant model; Hap = haplotype; KCND2 = potassium voltage-gated channel subfamily A member 1, KCNS1 = potassium voltage-gated channel modifier subfamily S member 1; KCNJ3 = potassium inwardly rectifying channel subfamily J member 3; KCNJ5 = potassium inwardly rectifying channel subfamily J member 5; KCNJ6 = potassium inwardly rectifying channel subfamily J member 6; KCNK2 = potassium two pore domain channel subfamily K member 2; KCNK9 = potassium two pore domain channel subfamily K member 9; MAF = minor allele frequency; R = recessive model; SNP= single nucleotide polymorphism.

**TABLE 2:** Multiple Logistic Regression Analyses for Single Nucleotide Polymorphisms in Potassium Channel Genes and the Occurrence of Palpitations in Women Prior to Breast Cancer Surgery

Predictor	Adjusted Odds Ratio	Standard Error	95% CI	Z	*p*-value

Potassium voltage-gated channels					
KCNS1 rs4499491	2.03	0.52	1.23, 3.36	2.77	.006
KPS score	0.98	0.01	0.95, 1.00	−1.72	.085
Occurrence of back pain	2.19	0.81	1.06, 4.51	2.12	.034
Overall model fit: X^2^ = 28.10; *p* = .0009; pseudo R^2^ = .1078					
Potassium inwardly rectifying channels					
KCNJ3 rs717175	2.49	0.94	1.19, 5.23	2.42	.016
KPS score	0.97	0.01	0.94, 0.99	−2.09	.036
Occurrence of back pain	2.18	0.81	1.05, 4.50	2.10	.035
Overall model fit: X2 = 26.42; *p* = .0017; pseudo R^2^ = .1013					
KCNJ5 rs11221510	5.68	3.11	1.95, 16.59	3.18	.001
KPS score	0.96	0.01	0.94, 0.99	−2.66	.008
Occurrence of back pain	2.30	0.85	1.11, 4.76	2.24	.025
Overall model fit: X2 = 29.08; *p* = .0006; pseudo R^2^ = .1115					
KCNJ6 rs13049947	2.73	1.22	1.14, 6.53	2.25	.025
KCNJ6 rs1399596	2.83	1.04	1.39, 5.80	2.85	.004
KPS score	0.97	0.01	0.94, 0.99	−2.13	.033
Occurrence of back pain	2.37	0.90	1.13, 4.99	2.28	.022
Overall model fit: X^2^ = 36.83; *p* = .0001; pseudo R^2^ = .1412					
Potassium two pore domain channels					
KCNK2 rs12757222	0.45	0.18	0.21, 0.99	−1.99	.046
KPS score	0.97	0.01	0.94, 0.99	−2.16	.031
Occurrence of back pain	2.20	0.81	1.07, 4.51	2.15	.031
Overall model fit: X^2^ = 24.31; *p* = .0038; pseudo R^2^ = .0932					

Note. Multiple logistic regression analyses of candidate gene associations with no palpitations versus palpitations. For each model, the first three principal components identified from the analysis of ancestry informative markers as well as self-report race/ethnicity were retained in all models to adjust for potential confounding due to race or ethnicity (data not shown). Predictors evaluated in each model included genotype (KCNS1 rs4499491: CC versus CA versus AA; KCNJ3 rs717175: CC versus CT + TT; KCNJ5 rs11221510: AA + AT versus TT; KCNJ6 rs13049947: CC versus CT + TT; KCNJ6 rs1399596: TT versus TC + CC; KCNK2 rs12757222: AA versus AG + GG), KPS score (in 10 unit increments), and self-reported occurrence of back pain.

CI = confidence interval; KCNS1 = potassium voltage-gated channel modifier subfamily S member 1; KCNJ3 = potassium inwardly rectifying channel subfamily J member 3; KCNJ5 = potassium inwardly rectifying channel subfamily J member 5; KCNJ6 = potassium inwardly rectifying channel subfamily J member 6; KCNK2 = potassium two pore domain channel subfamily K member 2; KPS = Karnofsky Performance Status.

## Layperson Summary

### What we investigated and why

A significant percentage of patients with breast cancer report palpitations (i.e., sensation of heart racing or pounding). However, the mechanisms that underlie this symptom are not well understood. This study evaluated for associations between the occurrence of palpitations and variations for potassium channel genes.

### How we did our research

Prior to breast cancer surgery, women reported whether or not they experienced palpitations in the past week and provided a blood sample for genomic analyses. Analyses were done to determine associations between the occurrence of palpitations and 155 different variations for 10 potassium channel genes.

### What we found

Significant associations were found between the occurrence of palpitations and six variations among five candidate genes.

### What it means

These findings suggest that variations in multiple potassium channel genes may play a role in the development of palpitations.
