# Supplementary material for: Associations Between Potassium Channel Genes and the Occurrence of Palpitations in Women Prior to Breast Cancer Surgery
Source: Semin Oncol Nurs. Author manuscript; Available in PMC 2026 Jul 9. (PMC13348194; doi:10.1016/j.soncn.2025.152039)
Supplement: 1 [file NIHMS2183339-supplement-1.docx]

Supplementary Table 1 - Summary of Single Nucleotide Polymorphisms Analyzed for Potassium Channel Genes Between the No Palpitations and Palpitations Groups

| *Gene* | *SNP* | *Position* | *Chr* | *MAF* | *Alleles* | *Chi Square* | *p-value* | *Model* |
| --- | --- | --- | --- | --- | --- | --- | --- | --- |
| POTASSIUM VOLTAGE-GATED CHANNELS | | | | | | | | |
| KCNA1 | rs4766311 | 4892699 | 1 | 0.466 | C>T | 0.419 | .811 | A |
| KCND2 | rs17376373 | 119787721 | 7 | 0.197 | T>G | 0.293 | .864 | A |
| KCND2 | rs702414 | 119924204 | 7 | 0.249 | G>C | 0.079 | .961 | A |
| KCND2 | rs802340 | 119975021 | 7 | 0.293 | G>T | 0.784 | .676 | A |
| KCND2 | rs12706292 | 120012310 | 7 | 0.346 | A>G | 0.872 | .647 | A |
| KCND2 | rs4730967 | 120060462 | 7 | 0.320 | T>C | FE | .039 | R |
| KCND2 | rs1072198 | 120114585 | 7 | 0.304 | A>G | 0.780 | .677 | A |
| KCND2 | rs11489533 | 120117902 | 7 | 0.268 | A>G | 12.364 | .002 | A |
| KCND2 | rs4727914 | 120122574 | 7 | 0.343 | A>G | 4.389 | .111 | A |
| KCND2 | rs12673992 | 120160059 | 7 | 0.319 | A>G | 1.962 | .375 | A |
| KCND2 | HapA1 |  |  |  |  | 12.435 | .002 |  |
| KCND2 | HapA3 |  |  |  |  | 4.389 | .111 |  |
| KCNS1 | rs4499491 | 43154833 | 20 | 0.432 | C>A | 6.747 | .034 | A |
| KCNS1 | rs6124684 | 43154907 | 20 | 0.223 | C>T | FE | .036 | D |
| KCNS1 | rs734784 | 43157041 | 20 | 0.447 | A>G | 0.265 | .876 | A |
| KCNS1 | rs6073643 | 43161484 | 20 | 0.274 | T>C | 2.507 | .285 | A |
| KCNS1 | HapA1 |  |  |  |  | 6.748 | .034 |  |
| KCNS1 | HapA2 |  |  |  |  | 3.636 | .162 |  |
| KCNS1 | HapA3 |  |  |  |  | 4.599 | .100 |  |
| KCNS1 | HapB1 |  |  |  |  | 0.265 | .876 |  |
| KCNS1 | HapB2 |  |  |  |  | 2.179 | .336 |  |
| KCNS1 | HapB3 |  |  |  |  | 2.457 | .293 |  |
| POTASSIUM INWARDLY RECTIFYING CHANNELS | | | | | | | | |
| KCNJ3 | rs6435329 | 155265893 | 2 | 0.445 | G>T | 0.935 | .627 | A |
| KCNJ3 | rs3111020 | 155275635 | 2 | 0.450 | T>C | 1.833 | .400 | A |
| KCNJ3 | rs11895478 | 155279369 | 2 | 0.246 | C>T | 1.876 | .391 | A |
| KCNJ3 | rs3106653 | 155283806 | 2 | 0.262 | A>C | 0.025 | .988 | A |
| KCNJ3 | rs3111003 | 155300413 | 2 | 0.465 | C>T | 2.142 | .343 | A |
| KCNJ3 | rs3111006 | 155302345 | 2 | 0.375 | C>T | 1.796 | .407 | A |
| KCNJ3 | rs12471193 | 155304383 | 2 | 0.343 | A>G | 2.470 | .291 | A |
| KCNJ3 | rs6711727 | 155304684 | 2 | 0.485 | G>A | 3.116 | .211 | A |
| KCNJ3 | rs2652443 | 155313983 | 2 | 0.395 | G>A | 0.404 | .817 | A |
| KCNJ3 | rs7574878 | 155315394 | 2 | 0.429 | T>G | 3.619 | .164 | A |
| KCNJ3 | rs2121085 | 155315711 | 2 | 0.447 | A>G | 2.798 | .247 | A |
| KCNJ3 | rs2121089 | 155317633 | 2 | 0.479 | C>A | 2.413 | .299 | A |
| KCNJ3 | rs2961959 | 155326068 | 2 | 0.432 | C>G | 0.904 | .636 | A |
| KCNJ3 | rs2591168 | 155326179 | 2 | 0.316 | A>G | 2.106 | .349 | A |
| KCNJ3 | rs2591172 | 155330423 | 2 | 0.333 | T>G | 2.191 | .334 | A |
| KCNJ3 | rs12995382 | 155340539 | 2 | 0.290 | T>C | 1.641 | .440 | A |
| KCNJ3 | rs13398937 | 155348593 | 2 | 0.362 | C>G | FE | .038 | D |
| KCNJ3 | rs13390038 | 155351011 | 2 | 0.403 | G>A | 1.437 | .487 | A |
| KCNJ3 | rs12616121 | 155353928 | 2 | 0.469 | A>G | 2.389 | .303 | A |
| KCNJ3 | rs2591158 | 155355912 | 2 | 0.280 | A>C | 2.230 | .328 | A |
| KCNJ3 | rs2591157 | 155356612 | 2 | 0.330 | A>G | 0.829 | .661 | A |
| KCNJ3 | rs717175 | 155356841 | 2 | 0.332 | C>T | FE | .040 | D |
| KCNJ3 | rs1037091 | 155360603 | 2 | 0.375 | G>A | 1.001 | .606 | A |
| KCNJ3 | rs17641121 | 155373998 | 2 | 0.259 | T>C | 3.856 | .145 | A |
| KCNJ3 | rs2591173 | 155395322 | 2 | 0.477 | C>A | 0.104 | .949 | A |
| KCNJ3 | rs2971902 | 155400624 | 2 | 0.220 | G>T | 0.326 | .850 | A |
| KCNJ3 | rs2937600 | 155411014 | 2 | 0.299 | A>G | 1.321 | .517 | A |
| KCNJ3 | rs4467223 | 155414657 | 2 | 0.479 | T>A | 5.670 | .059 | A |
| KCNJ3 | HapA1 |  |  |  |  | 7.113 | .029 |  |
| KCNJ3 | HapA2 |  |  |  |  | 1.876 | .391 |  |
| KCNJ3 | HapA3 |  |  |  |  | 1.833 | .400 |  |
| KCNJ3 | HapB1 |  |  |  |  | 1.387 | .500 |  |
| KCNJ3 | HapB4 |  |  |  |  | 0.085 | .958 |  |
| KCNJ3 | HapC3 |  |  |  |  | 0.134 | .935 |  |
| KCNJ3 | HapC5 |  |  |  |  | 1.714 | .424 |  |
| KCNJ3 | HapD1 |  |  |  |  | 3.309 | .191 |  |
| KCNJ3 | HapD4 |  |  |  |  | 0.261 | .878 |  |
| KCNJ3 | HapE1 |  |  |  |  | 1.463 | .481 |  |
| KCNJ3 | HapE2 |  |  |  |  | 0.248 | .883 |  |
| KCNJ3 | HapE4 |  |  |  |  | 2.981 | .225 |  |
| KCNJ3 | HapF1 |  |  |  |  | 1.011 | .603 |  |
| KCNJ3 | HapF2 |  |  |  |  | 0.358 | .836 |  |
| KCNJ3 | HapF4 |  |  |  |  | 2.233 | .327 |  |
| KCNJ3 | HapG1 |  |  |  |  | 1.294 | .524 |  |
| KCNJ3 | HapG3 |  |  |  |  | 0.344 | .842 |  |
| KCNJ3 | HapG4 |  |  |  |  | 0.084 | .959 |  |
| KCNJ5 | rs7941582 | 128266885 | 11 | 0.408 | A>G | 2.887 | .236 | A |
| KCNJ5 | rs2846700 | 128274148 | 11 | 0.172 | A>G | 1.987 | .370 | A |
| KCNJ5 | rs4937384 | 128285012 | 11 | 0.223 | T>C | 1.507 | .471 | A |
| KCNJ5 | rs11221503 | 128277662 | 11 | 0.184 | C>T | FE | .049 | R |
| KCNJ5 | rs2604212 | 128278165 | 11 | 0.459 | C>G | 1.952 | .377 | A |
| KCNJ5 | rs4937387 | 128278623 | 11 | 0.257 | T>C | 2.363 | .307 | A |
| KCNJ5 | rs11221510 | 128285907 | 11 | 0.241 | A>T | FE | .029 | R |
| KCNJ5 | rs6590357 | 128286549 | 11 | 0.163 | C>T | 0.783 | .676 | A |
| KCNJ5 | HapA1 |  |  |  |  | 1.904 | .386 |  |
| KCNJ5 | HapA2 |  |  |  |  | 3.147 | .207 |  |
| KCNJ5 | HapA5 |  |  |  |  | 4.450 | .108 |  |
| KCNJ6 | rs860795 | 37937160 | 21 | 0.208 | G>C | 0.761 | .683 | A |
| KCNJ6 | rs1709838 | 37941983 | 21 | 0.431 | C>A | 1.651 | .438 | A |
| KCNJ6 | rs10483038 | 37946641 | 21 | 0.279 | T>C | 0.192 | .908 | A |
| KCNJ6 | rs857967 | 37954006 | 21 | 0.197 | T>A | 1.799 | .407 | A |
| KCNJ6 | rs2835885 | 37961436 | 21 | 0.432 | T>G | 5.854 | .054 | A |
| KCNJ6 | rs858010 | 37987109 | 21 | 0.166 | G>A | 2.491 | .288 | A |
| KCNJ6 | rs1005546 | 37990742 | 21 | 0.450 | C>T | 4.228 | .121 | A |
| KCNJ6 | rs858003 | 37994854 | 21 | 0.197 | C>T | 1.268 | .530 | A |
| KCNJ6 | rs1709816 | 37999129 | 21 | 0.390 | G>T | 2.646 | .266 | A |
| KCNJ6 | rs13049947 | 38002710 | 21 | 0.403 | C>T | FE | .029 | D |
| KCNJ6 | rs2835914 | 38020720 | 21 | 0.347 | G>C | 1.789 | .409 | A |
| KCNJ6 | rs858035 | 38021061 | 21 | 0.344 | T>C | FE | .017 | D |
| KCNJ6 | rs13048511 | 38037731 | 21 | 0.468 | A>G | FE | .044 | R |
| KCNJ6 | rs2835925 | 38041173 | 21 | 0.176 | A>G | 2.799 | .247 | A |
| KCNJ6 | rs857989 | 38042001 | 21 | 0.115 | G>C | 6.146 | .046 | A |
| KCNJ6 | rs2835931 | 38043518 | 21 | 0.282 | C>T | 2.631 | .268 | A |
| KCNJ6 | rs1399596 | 38045382 | 21 | 0.260 | T>C | FE | .004 | D |
| KCNJ6 | rs2835942 | 38052778 | 21 | 0.303 | C>T | 0.906 | .636 | A |
| KCNJ6 | rs2835945 | 38057170 | 21 | 0.398 | G>A | 0.472 | .790 | A |
| KCNJ6 | rs1160350 | 38065897 | 21 | 0.494 | G>C | 1.832 | .400 | A |
| KCNJ6 | rs762145 | 38068188 | 21 | 0.366 | C>T | 1.276 | .528 | A |
| KCNJ6 | rs2226356 | 38075902 | 21 | 0.427 | C>T | 0.960 | .619 | A |
| KCNJ6 | rs1787337 | 38077824 | 21 | 0.494 | A>G | 2.633 | .268 | A |
| KCNJ6 | rs2835961 | 38083028 | 21 | 0.482 | G>A | 1.141 | .565 | A |
| KCNJ6 | rs2835976 | 38103779 | 21 | 0.385 | C>T | 2.977 | .226 | A |
| KCNJ6 | rs2835977 | 38104067 | 21 | 0.224 | G>A | 2.639 | .267 | A |
| KCNJ6 | rs2211842 | 38105403 | 21 | 0.376 | C>A | 2.058 | .357 | A |
| KCNJ6 | rs2211843 | 38106055 | 21 | 0.234 | G>T | 0.464 | .793 | A |
| KCNJ6 | rs2211845 | 38106371 | 21 | 0.447 | T>C | 0.369 | .832 | A |
| KCNJ6 | rs2835982 | 38110247 | 21 | 0.368 | C>A | 0.036 | .982 | A |
| KCNJ6 | rs2835983 | 38110476 | 21 | 0.304 | G>A | 2.185 | .335 | A |
| KCNJ6 | rs2835984 | 38110657 | 21 | 0.497 | A>T | 1.254 | .534 | A |
| KCNJ6 | rs3787835 | 38111440 | 21 | 0.455 | C>T | 3.189 | .203 | A |
| KCNJ6 | rs6517435 | 38117092 | 21 | 0.422 | G>A | 1.962 | .375 | A |
| KCNJ6 | rs2154556 | 38120757 | 21 | 0.344 | T>C | FE | .025 | R |
| KCNJ6 | rs4817896 | 38123831 | 21 | 0.248 | C>T | 3.442 | .179 | A |
| KCNJ6 | rs3787840 | 38124263 | 21 | 0.139 | C>T | 1.136 | .567 | A |
| KCNJ6 | rs991985 | 38128024 | 21 | 0.286 | C>A | 3.351 | .187 | A |
| KCNJ6 | rs2836007 | 38128761 | 21 | 0.194 | C>T | 1.529 | .466 | A |
| KCNJ6 | rs2836013 | 38132582 | 21 | 0.292 | C>T | 2.162 | .339 | A |
| KCNJ6 | rs2836016 | 38134890 | 21 | 0.411 | A>G | 4.181 | .124 | A |
| KCNJ6 | rs2836019 | 38136864 | 21 | 0.327 | C>T | 0.383 | .826 | A |
| KCNJ6 | rs915800 | 38138203 | 21 | 0.455 | C>T | 3.372 | .185 | A |
| KCNJ6 | rs2226741 | 38146803 | 21 | 0.147 | A>G | 2.662 | .264 | A |
| KCNJ6 | rs7276928 | 38147607 | 21 | 0.288 | G>A | 0.759 | .684 | A |
| KCNJ6 | rs3827199 | 38149472 | 21 | 0.408 | G>A | 1.528 | .466 | A |
| KCNJ6 | rs4816585 | 38151120 | 21 | 0.495 | G>A | 0.442 | .802 | A |
| KCNJ6 | rs9305628 | 38166861 | 21 | 0.227 | A>G | 1.176 | .555 | A |
| KCNJ6 | rs9974219 | 38168568 | 21 | 0.277 | A>T | 0.075 | .963 | A |
| KCNJ6 | rs7277957 | 38168770 | 21 | 0.492 | A>G | 0.261 | .878 | A |
| KCNJ6 | rs1892682 | 38169935 | 21 | 0.265 | G>A | 1.869 | .393 | A |
| KCNJ6 | rs928765 | 38173472 | 21 | 0.292 | C>T | 0.803 | .669 | A |
| KCNJ6 | rs3787862 | 38174571 | 21 | 0.197 | G>A | 0.670 | .715 | A |
| KCNJ6 | rs10775660 | 38175388 | 21 | 0.415 | C>T | 0.796 | .672 | A |
| KCNJ6 | rs8129919 | 38176410 | 21 | 0.471 | G>A | 0.744 | .689 | A |
| KCNJ6 | rs2836039 | 38188930 | 21 | 0.195 | G>A | n/a | n/a | n/a |
| KCNJ6 | rs2836048 | 38206168 | 21 | 0.321 | G>A | 0.154 | .926 | A |
| KCNJ6 | rs2836050 | 38206705 | 21 | 0.227 | C>T | 0.045 | .978 | A |
| KCNJ6 | rs3787870 | 38207323 | 21 | 0.463 | A>G | 2.664 | .264 | A |
| KCNJ6 | HapA1 |  |  |  |  | 0.566 | .754 |  |
| KCNJ6 | HapA2 |  |  |  |  | 1.775 | .412 |  |
| KCNJ6 | HapA3 |  |  |  |  | 0.778 | .678 |  |
| KCNJ6 | HapB1 |  |  |  |  | 2.379 | .304 |  |
| KCNJ6 | HapB2 |  |  |  |  | 1.799 | .407 |  |
| KCNJ6 | HapB3 |  |  |  |  | 0.192 | .908 |  |
| KCNJ6 | HapC1 |  |  |  |  | 4.228 | .121 |  |
| KCNJ6 | HapC2 |  |  |  |  | 1.563 | .458 |  |
| KCNJ6 | HapC3 |  |  |  |  | 2.491 | .288 |  |
| KCNJ6 | HapD1 |  |  |  |  | 6.364 | .042 |  |
| KCNJ6 | HapD4 |  |  |  |  | 2.248 | .325 |  |
| KCNJ6 | HapD6 |  |  |  |  | 1.145 | .564 |  |
| KCNJ6 | HapE1 |  |  |  |  | 8.793 | .012 |  |
| KCNJ6 | HapE2 |  |  |  |  | 2.719 | .257 |  |
| KCNJ6 | HapE5 |  |  |  |  | 2.621 | .270 |  |
| KCNJ6 | HapE7 |  |  |  |  | 2.898 | .235 |  |
| KCNJ6 | HapF1 |  |  |  |  | 0.984 | .611 |  |
| KCNJ6 | HapF2 |  |  |  |  | 1.020 | .600 |  |
| KCNJ6 | HapF4 |  |  |  |  | 1.094 | .579 |  |
| KCNJ6 | HapG1 |  |  |  |  | 1.168 | .558 |  |
| KCNJ6 | HapG5 |  |  |  |  | 5.186 | .075 |  |
| KCNJ6 | HapG6 |  |  |  |  | 2.470 | .291 |  |
| KCNJ6 | HapH1 |  |  |  |  | 3.938 | .140 |  |
| KCNJ6 | HapH3 |  |  |  |  | 0.076 | .963 |  |
| KCNJ6 | HapH5 |  |  |  |  | 0.472 | .790 |  |
| KCNJ6 | HapI1 |  |  |  |  | 4.724 | .094 |  |
| KCNJ6 | HapI5 |  |  |  |  | 3.423 | .181 |  |
| KCNJ6 | HapJ1 |  |  |  |  | 1.478 | .478 |  |
| KCNJ6 | HapJ2 |  |  |  |  | 2.162 | .339 |  |
| KCNJ6 | HapJ3 |  |  |  |  | 1.531 | .465 |  |
| KCNJ6 | HapK1 |  |  |  |  | 3.372 | .185 |  |
| KCNJ6 | HapK4 |  |  |  |  | 0.359 | .836 |  |
| KCNJ6 | HapL1 |  |  |  |  | 0.168 | .919 |  |
| KCNJ6 | HapL4 |  |  |  |  | 0.266 | .875 |  |
| KCNJ6 | HapL5 |  |  |  |  | 1.157 | .561 |  |
| KCNJ6 | HapM1 |  |  |  |  | 0.818 | .664 |  |
| KCNJ6 | HapM4 |  |  |  |  | 0.591 | .744 |  |
| KCNJ6 | HapM6 |  |  |  |  | 0.857 | .652 |  |
| KCNJ6 | HapN2 |  |  |  |  | 0.057 | .972 |  |
| KCNJ6 | HapN3 |  |  |  |  | 0.226 | .893 |  |
| KCNJ9 | rs6677510 | 158318743 | 1 | 0.442 | A>G | 0.149 | .928 | A |
| KCNJ9 | rs2753268 | 158324876 | 1 | 0.260 | C>T | 1.041 | .594 | A |
| POTASSIUM TWO PORE DOMAIN CHANNELS | | | | | | | | |
| KCNK2 | rs2601640 | 213253979 | 1 | 0.492 | A>G | 3.748 | .154 | A |
| KCNK2 | rs12141327 | 213273537 | 1 | 0.335 | G>A | 1.031 | .597 | A |
| KCNK2 | rs1452619 | 213280153 | 1 | 0.120 | A>G | 3.482 | .175 | A |
| KCNK2 | rs10494991 | 213287222 | 1 | 0.331 | T>C | 3.510 | .173 | A |
| KCNK2 | rs1584759 | 213289445 | 1 | 0.453 | A>C | 2.276 | .321 | A |
| KCNK2 | rs12064317 | 213293664 | 1 | 0.136 | G>T | 0.011 | .995 | A |
| KCNK2 | rs6665177 | 213298091 | 1 | 0.155 | G>A | 0.866 | .649 | A |
| KCNK2 | rs12028008 | 213298169 | 1 | 0.497 | A>G | 2.168 | .338 | A |
| KCNK2 | rs12038094 | 213302819 | 1 | 0.291 | C>T | 0.491 | .782 | A |
| KCNK2 | rs17024179 | 213304166 | 1 | 0.163 | T>C | 0.250 | .882 | A |
| KCNK2 | rs7528988 | 213315040 | 1 | 0.259 | C>T | 2.874 | .249 | A |
| KCNK2 | rs2363561 | 213321930 | 1 | 0.395 | C>T | 2.060 | .357 | A |
| KCNK2 | rs12133857 | 213331109 | 1 | 0.128 | G>T | 0.999 | .607 | A |
| KCNK2 | rs4411107 | 213355542 | 1 | 0.375 | T>C | 3.109 | .211 | A |
| KCNK2 | rs4303048 | 213385781 | 1 | 0.236 | G>A | 2.340 | .310 | A |
| KCNK2 | rs12757222 | 213391641 | 1 | 0.233 | A>G | FE | .024 | D |
| KCNK2 | rs1556905 | 213428215 | 1 | 0.411 | C>A | 1.040 | .594 | A |
| KCNK2 | rs10494994 | 213428830 | 1 | 0.207 | G>A | 0.400 | .819 | A |
| KCNK2 | rs12038695 | 213444580 | 1 | 0.494 | A>C | 2.141 | .343 | A |
| KCNK2 | rs2027320 | 213446566 | 1 | 0.385 | G>A | 1.967 | .374 | A |
| KCNK2 | rs12143625 | 213458463 | 1 | 0.235 | T>C | 4.498 | .105 | A |
| KCNK2 | rs12080135 | 213463166 | 1 | 0.252 | T>G | 1.302 | .522 | A |
| KCNK2 | HapA1 |  |  |  |  | 1.031 | .597 |  |
| KCNK2 | HapA4 |  |  |  |  | 3.748 | .154 |  |
| KCNK2 | HapB1 |  |  |  |  | 2.140 | .343 |  |
| KCNK2 | HapB4 |  |  |  |  | 3.070 | .215 |  |
| KCNK2 | HapC1 |  |  |  |  | 2.931 | .231 |  |
| KCNK2 | HapC4 |  |  |  |  | 0.157 | .925 |  |
| KCNK2 | HapC5 |  |  |  |  | 2.168 | .338 |  |
| KCNK2 | HapD1 |  |  |  |  | 2.606 | .272 |  |
| KCNK2 | HapD3 |  |  |  |  | 2.060 | .357 |  |
| KCNK2 | HapE1 |  |  |  |  | 0.764 | .683 |  |
| KCNK2 | HapE3 |  |  |  |  | 1.904 | .386 |  |
| KCNK2 | HapE4 |  |  |  |  | 0.020 | .990 |  |
| KCNK2 | HapF2 |  |  |  |  | 1.967 | .374 |  |
| KCNK2 | HapF3 |  |  |  |  | 2.141 | .343 |  |
| KCNK3 | rs1275982 | 26772593 | 2 | 0.497 | C>T | 1.645 | .439 | A |
| KCNK3 | rs1275977 | 26776359 | 2 | 0.414 | A>G | 0.186 | .911 | A |
| KCNK3 | rs11126666 | 26782315 | 2 | 0.330 | G>A | 0.155 | .926 | A |
| KCNK3 | rs1662987 | 26791686 | 2 | 0.243 | A>G | 1.470 | .480 | A |
| KCNK3 | rs1662988 | 26793738 | 2 | 0.290 | C>T | 0.679 | .712 | A |
| KCNK3 | rs7584568 | 26798797 | 2 | 0.471 | G>A | 0.148 | .928 | A |
| KCNK3 | HapA1 |  |  |  |  | 0.155 | .926 |  |
| KCNK3 | HapA4 |  |  |  |  | 1.645 | .439 |  |
| KCNK3 | HapB1 |  |  |  |  | 0.165 | .921 |  |
| KCNK3 | HapB2 |  |  |  |  | 0.945 | .623 |  |
| KCNK3 | HapB4 |  |  |  |  | 0.698 | .705 |  |
| KCNK9 | rs2542424 | 140701683 | 8 | 0.362 | A>G | 1.395 | .498 | A |
| KCNK9 | rs2542422 | 140706306 | 8 | 0.328 | C>A | 1.934 | .380 | A |
| KCNK9 | rs2014712 | 140709816 | 8 | 0.235 | C>T | 1.516 | .468 | A |
| KCNK9 | rs2545462 | 140714686 | 8 | 0.343 | C>A | 1.216 | .544 | A |
| KCNK9 | rs2542420 | 140714883 | 8 | 0.419 | C>G | 0.411 | .814 | A |
| KCNK9 | rs2545461 | 140717431 | 8 | 0.257 | A>G | n/a | n/a | n/a |
| KCNK9 | rs3780051 | 140727983 | 8 | 0.471 | A>G | 1.120 | .571 | A |
| KCNK9 | rs2545457 | 140730467 | 8 | 0.350 | T>C | 0.291 | .865 | A |
| KCNK9 | rs2005895 | 140738217 | 8 | 0.256 | T>C | 2.168 | .338 | A |
| KCNK9 | rs888349 | 140738927 | 8 | 0.197 | A>C | 0.955 | .620 | A |
| KCNK9 | rs759656 | 140739149 | 8 | 0.320 | T>C | n/a | n/a | n/a |
| KCNK9 | rs13277242 | 140739269 | 8 | 0.495 | G>A | 0.093 | .955 | A |
| KCNK9 | rs885724 | 140740112 | 8 | 0.380 | A>C | 2.249 | .325 | A |
| KCNK9 | rs3780039 | 140745846 | 8 | 0.372 | T>G | 2.115 | .347 | A |
| KCNK9 | rs10110946 | 140754803 | 8 | 0.333 | T>C | 1.965 | .374 | A |
| KCNK9 | rs7828107 | 140756023 | 8 | 0.409 | C>A | 0.960 | .619 | A |
| KCNK9 | rs983740 | 140762922 | 8 | 0.472 | T>G | 0.583 | .747 | A |
| KCNK9 | rs11166921 | 140776937 | 8 | 0.395 | C>A | 0.460 | .795 | A |
| KCNK9 | rs13278664 | 140779544 | 8 | 0.455 | A>G | 0.622 | .733 | A |
| KCNK9 | HapA1 |  |  |  |  | 3.952 | .139 |  |
| KCNK9 | HapA2 |  |  |  |  | 1.826 | .401 |  |
| KCNK9 | HapA3 |  |  |  |  | 1.468 | .480 |  |
| KCNK9 | HapB1 |  |  |  |  | 0.558 | .756 |  |
| KCNK9 | HapB4 |  |  |  |  | 0.917 | .632 |  |
| KCNK9 | HapC1 |  |  |  |  | 1.837 | .399 |  |
| KCNK9 | HapC3 |  |  |  |  | 1.135 | .567 |  |
| KCNK9 | HapC4 |  |  |  |  | 0.841 | .657 |  |
| KCNK9 | HapD1 |  |  |  |  | 6.916 | .031 |  |
| KCNK9 | HapD2 |  |  |  |  | 0.952 | .621 |  |
| KCNK9 | HapD3 |  |  |  |  | 0.607 | .738 |  |

Abbreviations: A = additive model; Chr = chromosome; D = dominant model; Hap = haplotype; KCNA1 = potassium voltage-gated channel subfamily A member 1; KCND2 = potassium voltage-gated channel subfamily D member 2; KCNS1 = potassium voltage-gated channel modifier subfamily S member 1; KCNJ3 = potassium inwardly rectifying channel subfamily J member 3; KCNJ5 = potassium inwardly rectifying channel subfamily J member 5; KCNJ6 = potassium inwardly rectifying channel subfamily J member 6; KCNJ9 = potassium inwardly rectifying channel subfamily J member 9; KCNK2 = potassium two pore domain channel subfamily K member 2; KCNK3 = potassium two pore domain channel subfamily K member 3; KCNK9 = potassium two pore domain channel subfamily K member 9; MAF = minor allele frequency; n/a = not assayed because SNP violated Hardy-Weinberg expectations (p<.001) or because MAF was <.05; R = recessive model; SNP= single nucleotide polymorphism
