# Supplementary material for: Associations Between Potassium Channel Genes and the Occurrence of Palpitations in Women Prior to Breast Cancer Surgery
Source: Semin Oncol Nurs. Author manuscript; Available in PMC 2026 Jul 9. (PMC13348194; doi:10.1016/j.soncn.2025.152039)
Supplement: 2 [file NIHMS2183339-supplement-2.docx]

Supplementary Table 2 - Differences in Demographic and Clinical Characteristics Between the No Palpitations and Palpitations Groups Prior to Breast Cancer Surgery

| Characteristic | No Palpitations Group  (n=338) 84.9% | | Palpitations Group  (n=60) 15.1% | | Statistic | p-value |
| --- | --- | --- | --- | --- | --- | --- |
|  | Mean | SD | Mean | SD |  |  |
| Age (years) | 55.0 | 11.7 | 54.5 | 10.7 | t=0.30 | 0.768 |
| Education (years) | 15.7 | 2.7 | 15.7 | 2.6 | t=0.08 | 0.936 |
| Body mass index (kilograms/meter squared) | 26.5 | 5.8 | 28.5 | 7.7 | t=-1.97 | 0.052 |
| Karnofsky Performance Status score | 93.9 | 10.0 | 89.7 | 11.1 | t=2.91 | 0.004 |
| Self-administered Comorbidity Questionnaire score | 4.1 | 2.7 | 5.3 | 3.2 | t=-2.93 | 0.004 |
| Number of breast biopsies in past year | 1.5 | 0.8 | 1.5 | 0.8 | t=0.39 | 0.698 |
|  | n | % | n | % |  |  |
| Ethnicity  White  Black  Asian/Pacific Islander  Hispanic/Mixed ethnic background/Other | 216  32  41  47 | 64.3  9.5  12.2  14.0 | 39  8  9  4 | 65.0  13.3  15.0  6.7 | X^2^=3.17 | 0.366 |
| Married/partnered (% yes) | 140 | 41.8 | 25 | 42.4 | FE | 1.000 |
| Lives alone (% yes) | 76 | 22.8 | 19 | 32.2 | FE | 0.137 |
| Work for pay (% yes) | 160 | 47.6 | 29 | 49.2 | FE | 0.888 |
| Annual household income  <$30,000  $30,000 - $99,000  >$100,000 | 53  111  112 | 19.2  40.2  40.6 | 17  23  13 | 32.1  43.4  24.5 | U | 0.001 |
| Comorbid conditions (% yes)  Heart disease  High blood pressure  Lung disease  Diabetes  Ulcer  Kidney disease  Liver disease  Anemia  Depression  Osteoarthritis  Back pain  Rheumatoid arthritis | 10  99  9  23  13  3  9  25  69  54  86  11 | 3.0  29.3  2.7  6.8  3.8  0.9  2.7  7.4  20.4  16.0  25.4  3.3 | 5  24  3  8  2  0  1  7  18  15  26  3 | 8.3  40.0  5.0  13.3  3.3  0.0  1.7  11.7  30.0  25.0  43.3  5.0 | FE  FE  FE  FE  FE  FE  FE  FE  FE  FE  FE  FE | 0.059  0.129  0.402  0.111  1.000  1.000  1.000  0.299  0.126  0.097  0.008  0.452 |
| Stage of disease  0  I  IIA and IIB  IIIA, IIIB, IIIC, and IV | 63  132  117  26 | 18.6  39.1  34.6  7.7 | 10  19  24  7 | 16.7  31.7  40.0  11.7 | U | 0.205 |
| Estrogen receptor positive (% yes) | 257 | 76.3 | 50 | 83.3 | FE | 0.315 |
| Progesterone receptor positive (% yes) | 237 | 70.3 | 42 | 70.0 | FE | 1.000 |
| HER2/neu receptor positive (% yes) | 47 | 15.4 | 12 | 22.6 | FE | 0.227 |
| Received neoadjuvant chemotherapy (% yes) | 63 | 18.7 | 16 | 26.7 | FE | 0.162 |
| On hormone replacement therapy prior to the diagnosis of breast cancer (% yes) | 55 | 16.3 | 12 | 20.3 | FE | 0.453 |
| Gone through menopause (% yes) | 210 | 63.8 | 38 | 65.5 | FE | 0.882 |

FE – Fisher’s Exact test; SD – standard deviation; U – Mann Whitney U test
